# Supplementary material for: An Adapted GeneSwitch Toolkit for Comparable Cellular and Animal Models: A Proof of Concept in Modeling Charcot-Marie-Tooth Neuropathy
Source: Int J Mol Sci. 2023 Nov 9;24(22):16138. doi: 10.3390/ijms242216138 (PMC10670994; doi:10.3390/ijms242216138)
Supplement: Supplementary file 1 [file ijms-24-16138-s001.zip › ijms-2665991-supplementary.pdf]

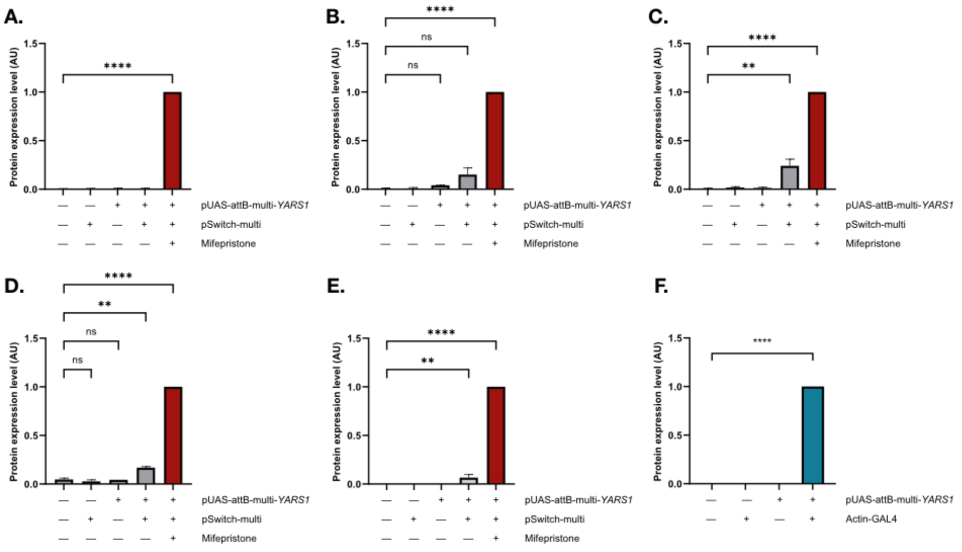

Figure S1. Quantification of YARS1 exogenous signal in (A) N2a, **(B)** HeLa, **(C)** CHO-K1, **(D)** HEK293T, **(E)** S2 cells. For each western blot presented in Figure 2, the relative quantification of band intensities of the HA-tagged YARS1 exogenous signal in absence of Mifepristone (grey) compared to the condition in response to Mifepristone (red) is presented. **(F)** The relative quantification of band intensities of YARS1 exogenous signal (grey) compared to the condition in presence of an Actin-GAL4 driver (blue) in S2 cells is depicted. Statistical significance (**P<0.01, ****P<0.0001, ns – not significant) was determined after one-way Anova analysis (n=3).

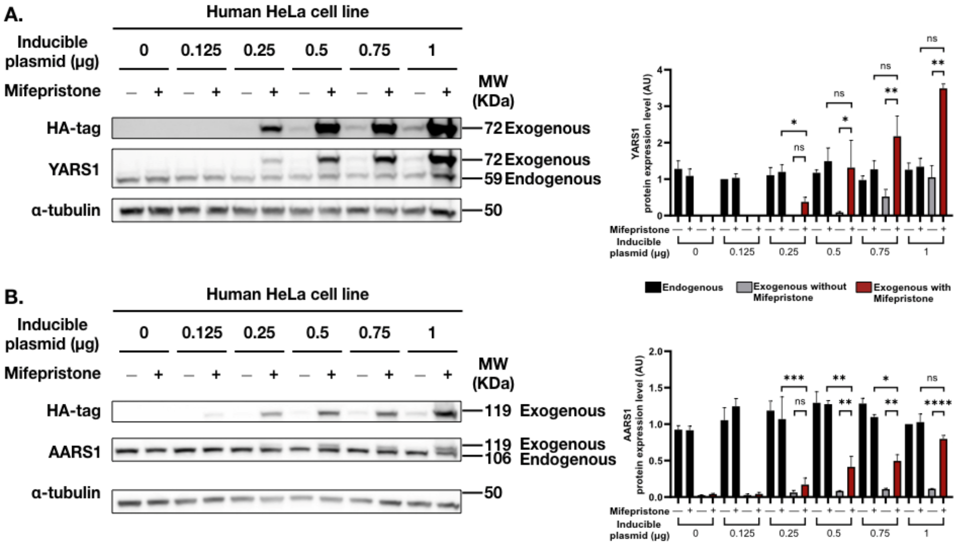

Figure S2. Inducible plasmid concentration to be transfected in HeLa cell line stably expressing the pSwitch-multi regulatory vector. HeLa stable cells for the pSwitch-multi regulatory vector have been transiently transfected with increased concentration of inducible plasmid (ranging from 0 to 1μg) containing (A) YARS1 or (B) AARS1 cDNA sequence. Four hours after transfection, 10nM of Mifepristone was added to the medium to induce transgene expression for 24h. Transgene signal was first determined by using HA-tag antibody. Exogenous and endogenous YARS1 and AARS1 expression was detected with mouse-monoclonal YARS1 or AARS1 antibodies, respectively. Equal loading was validated by using mouse-monoclonal α-tubulin antibody. Each graph represents the relative quantification of each band's intensity compared to the expression level at 0.5 μg inducible plasmid without the presence of Mifepristone (n=3). Bar charts are represented with s.e.m. for endogenous (black), exogenous in absence (grey) or presence of Mifepristone (red) YARS1 and AARS1, respectively. Statistical significance (*P<0.1, **P<0.01, ***P<0.001, ****P<0.0001, ns – not significant) was determined using one-way Anova analysis.

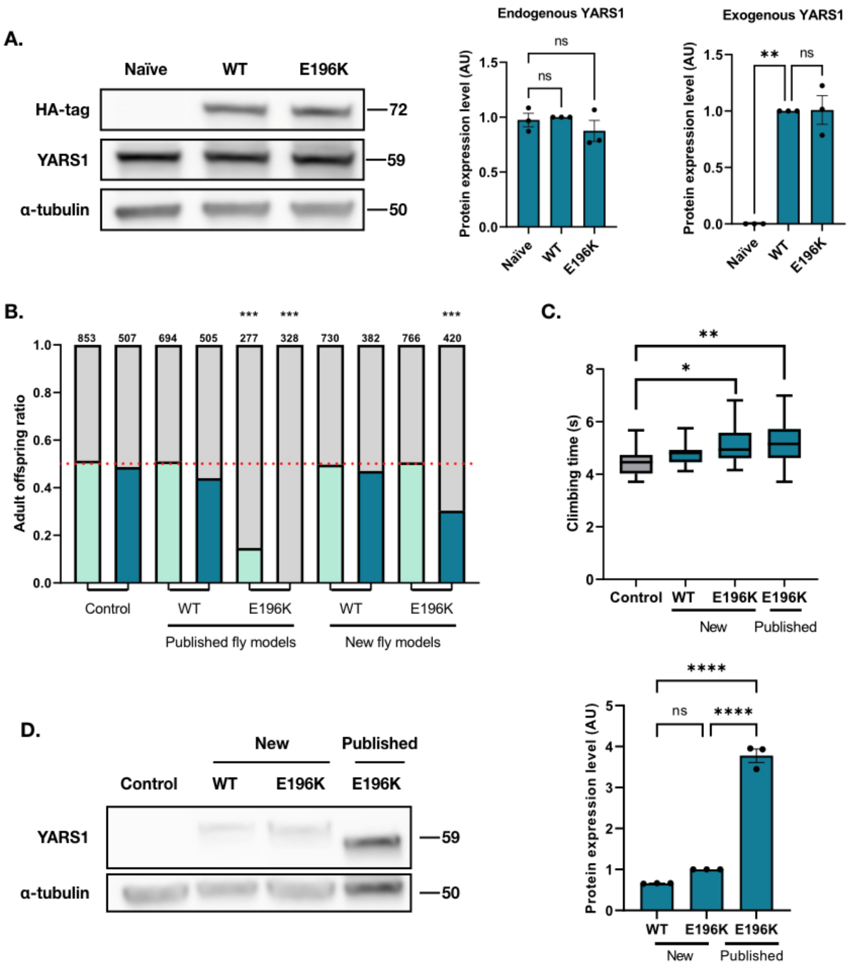

Figure S3. Characterization of YARS^{CMT} fly models. (A) Western blot analysis of protein extract from N2a cells expressing YARS1. Expression of exogenous and endogenous YARS1 were detected using mouse-monoclonal HA-tag and YARS1 antibody, respectively. Equal loading was validated by using mouse-monoclonal α-tubulin antibody (n=3). Graph representing relative quantification of exogenous and endogenous YARS1 bands. **(B)** Strong ubiquitous expression of YARS1^{WT} has no effect with Actin5c-GAL4weak (light blue) and Actin5c-GAL4strong (dark blue) on the expected 1:1 adult flies eclosion ratio. YARS1^{E196K} ubiquitous expression has detrimental effects in a dosage dependent manner in both new and previously published models. Yw flies were used as a negative control. The number of adult flies ecloding is indicated above each graph bar. Dashed line marks the expected 1:1 genotypes' eclosion ratio. Statistical significance (*P<0.1; ***P<0.0001; ****P<0,00001) was determined after One-Way Anova analysis which compares the odd ratios (Actin5c-GAL4/transgene ON - blue vs. Balancer/transgene OFF - grey) of flies in three independent crosses. **(C)** Panneuronal expression of mutant YARS1 with nSyb-GAL4 induces locomotor performance deficits as determined in a negative geotaxis climbing assay. NSyb-GAL4 flies were used as a negative control. The Y-axis indicates the time needed for the fastest fly to climb a vertical wall to a height of 82 mm. **P<0.01 was determined after one-way Anova analysis. Both lethality assay and negative geotaxis assay were performed on flies carrying the transgenic construct on the 3rd chromosome. **(D)** Western blot analysis of protein extract from newly and published YARS1^{CMT} fly models. Expression of exogenous YARS1 was detected using mouse

monoclonal YARS1 antibody. Equal loading was validated by using mouse-monoclonal α-tubulin antibody (n=3). Each graph representing relative quantification of YARS1 band intensities: bar charts are presented as means with s.e.m.; statistical significance (**P<0.01, ***P<0.001, ns – not significant) was determined after one-way Anova analysis (n=3).
